# Myocarditis and COVID-19 mRNA vaccines: a mechanistic hypothesis involving dsRNA

**DOI:** 10.2217/fvl-2021-0280

**Published:** 2021-12-06

**Authors:** Gerard Milano, Jocelyn Gal, Anne Creisson, Emmanuel Chamorey

**Affiliations:** ^1^Centre Antoine Lacassagne, Unité Propre de Recherche 7497, Université Côte d’Azur, 06100, Nice, France; ^2^Centre Antoine Lacassagne, UNS EA 7497 Nice University, 33 Avenue de Valombrose, 06189, Nice, France; ^3^Epidemiology & Biostatistics Department, Centre Antoine Lacassagne, University Côte d’Azur, 33 Avenue de Valombrose, 06189, Nice, France; ^4^Medical oncology Department, Centre Antoine Lacassagne, University Côte d’Azur, 33 Avenue de Valombrose, 06189, Nice, France

**Keywords:** COVID-19, dsRNA, mRNA COVID-19 vaccines, myocarditis

## Abstract

While tolerance to COVID-19 vaccination is considered satisfactory, a phenomenon of myocarditis, although rare, is becoming a safety concern in mRNA COVID-19 vaccination. The presence of low residual levels of double-strand RNA (dsRNA) has been reported in mRNA COVID-19 vaccine preparations. dsRNA is a known inducer of immune-inflammatory reactions. dsRNA present in vaccine nanoparticles may be suspected to be at the origin of the still unexplained cases of myocarditis.

Following the onset of the pandemic of SARS-CoV-2 (COVID-19), candidate vaccines have been developed at a particularly accelerated rate and with a remarkable efficacy. The SARS-CoV-2 Spike (S) protein is the key protein for the virus entry. Based on germinal genetic data [1] and strengthened by recent genome-wide CRISPR screen data [2], this protein has been found to specifically interact with its cellular target, the angiotensin-converting enzyme 2 (ACE2). The S protein, as the target of the majority of COVID-19 vaccines, has shown a significant capacity for mutations thus impacting vaccination [3–5]. To date, health authorities have authorized three main types of vaccines against SARS-CoV-2: vaccines that transfer nucleotide as DNA coding for the S protein (e.g., the AstraZeneca Vaxzevria vaccine [AZD1222]); the Jonhson & Jonhson (Janssen) vaccine (JNJ-78436735) and the Sputnick V vaccine and thereby introduce antigen-coding sequences within the DNA carried by the adenovirus; vaccines that deliver the S protein through engineered mRNA encapsulated in lipid nanoparticles (e.g., Pfizer BioNTech [BNT162b2] and Moderna [mRNA-1273]); and the inactivated Sinovac-CoronaVac (COVID-19) vaccine [6].

## The occurrence of myocarditis under COVID-19 vaccination

While tolerance to COVID-19 vaccination is considered satisfactory, a phenomenon of myocarditis, although rare, is becoming a safety concern in mRNA COVID-19 vaccination [7,8]. Myocarditis is an inflammatory phenomenon of the heart muscle; it is associated with pericarditis, an inflammation more specifically related to the pericardium, which is designed as myopericarditis. In the following text, the term myocarditis will be used to design myocarditis, pericarditis or myopericarditis. The highest rates of myocarditis were initially reported in young male adults [9]. In the reported cases, COVID-19 infection was ruled out and none of the patients had clinical signs or laboratory findings compatible with an autoimmune disease. Although in some instances histologically confirmed, these cases of myocarditis remain of unknown origin and are considered as possible adverse reactions following vaccination. Some investigators consider that host inflammatory cell responses are at the origin of these cases of myocarditis. Clearly, inflammation of the myocardium involving macrophage and dendritic cells plays a key role in triggering myocarditis in general [10]. Based on the medical reports of a group of 2,000,287 subjects who received at least one dose of COVID-19 vaccination, Diaz *et al.* [11] indicated that two distinct myocarditis syndromes could be observed after vaccination: first, an occurrence of myocarditis rapidly installed in younger subjects and mostly following the second dose; and second, pericarditis affecting older patients with a later occurrence after either the first or the second dose. More precisely, the report by Diaz *et al.* pointed that myocarditis and pericarditis occurred at the respective frequencies of 1.0 per 100,000 and 1.8 per 100,000 [11]. However, association does not prove causation and more investigations thus appear necessary at the clinical level not only to determine more precisely the incidence but also to establish the risk factors and treatment strategies in order to evaluate the long-term impact of vaccination-related myocarditis. In addition, more investigations are needed to elucidate the exact mechanisms triggering these mRNA COVID-19 vaccine-related cases of myocarditis. In this context, the US FDA has added information about the occurrence of these vaccine-related myocarditis cases to the Pfizer-BioNTech and Moderna COVID-19 vaccine emergency use authorizations and fact sheets [9]. The present analysis thus focused on mRNA vaccine-related myocarditis. As underlined in a recently issued editorial by Shay *et al.* [12], myocarditis occurrence following other vaccines is rare and has also been reported in conjunction with smallpox vaccination.

## Double-strand RNA as an impurity in mRNA vaccines

Before approving drugs and health products, health authorities deliver key authorizations usually based on publicly available reports. This was the case recently with the EMA regarding COVID-19 mRNA vaccines [13,14]. The EMA reported the presence in the Comirnaty and Moderna vaccine preparations of low residual levels of double-strand RNA (dsRNA) [13,14], which is one of the main impurities produced during mRNA vaccine preparation [15]. One of the characteristics of mRNA liposomal forms of vaccines is to be entirely produced *in vitro*. Thus, mRNA vaccines avoid the risks associated with other vaccine platforms, including live viruses, viral vectors, inactivated viruses and subunit protein vaccines. While the simplicity of the approach of synthesizing *in vitro*-transcribed (IVT) mRNA is appealing, technical difficulties remain, including impurities and particularly the generation of dsRNA contaminants [16] as indicated above. The current methods used to purify IVT mRNA vaccine preparations vary in terms of technical performance and, at best, allow the removal of 90% of dsRNA when using HPLC, as reported by the developers of mRNA vaccines [17]. According to reports and vaccine developers, the presence of short segments of dsRNA at low level along with purified mRNA cannot be totally ruled out [17].

## dsRNA & immune-inflammatory reactions

**Figure 1.**
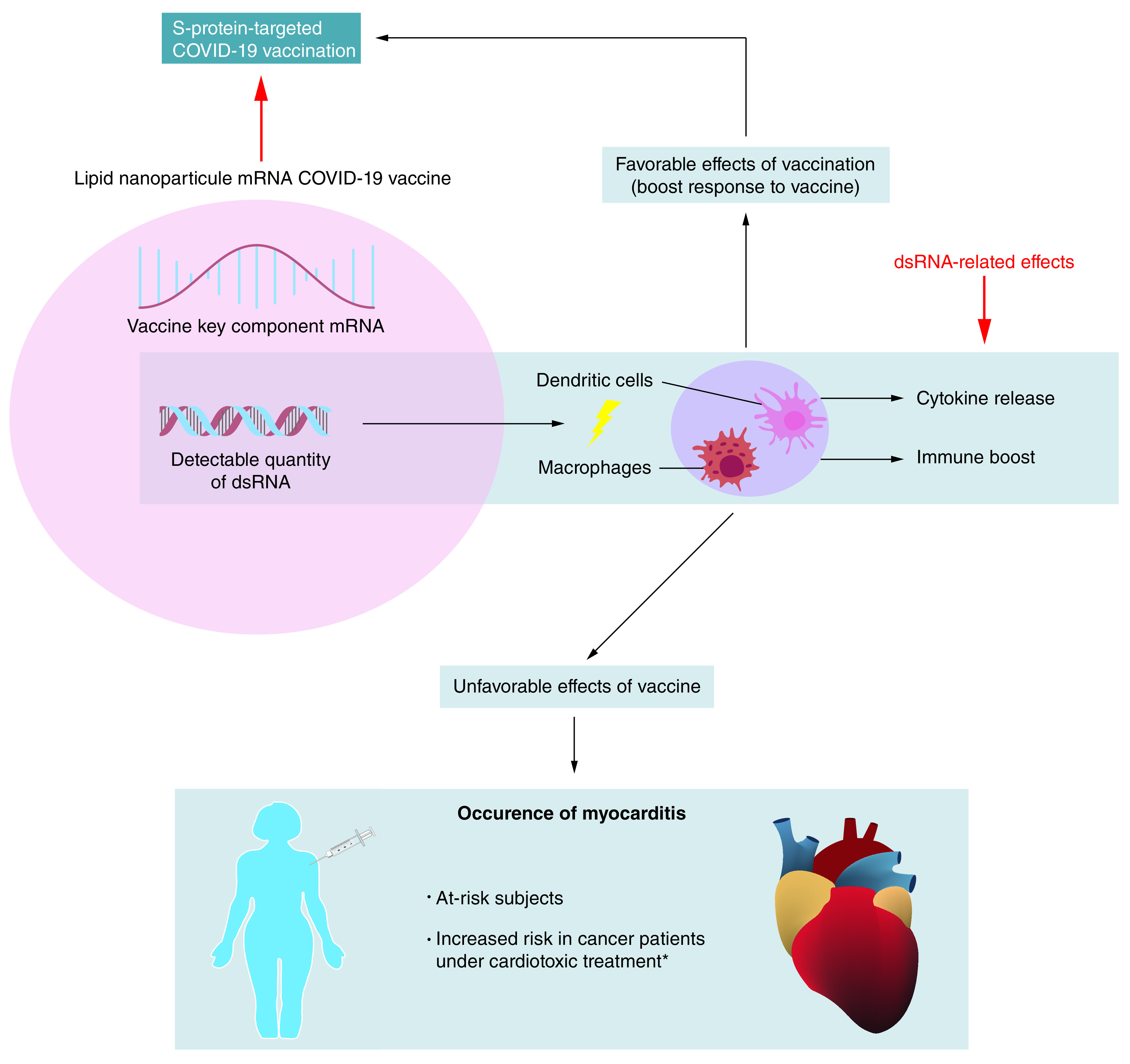
Myocarditis under the scope of mRNA COVID-19 vaccination. Double-strand RNA in mRNA COVID-19 vaccine preparations may induce a strong inflammatory response, which can favorably boost the expected response to vaccine. Double-strand RNA may also have possible unfavorable consequences like the development of myocarditis. *Mostly radiotherapy and chemotherapy by anthracyclines.

Notably, dsRNA is known to be a strong exogenous inducer of immune-inflammatory reactions involving well-identified intracellular signaling cascades and mediators (Figure 1). dsRNA is detected by antigen-presenting cells, endothelial cells and the airway epithelium [18], and gives rise to dose-related innate immune activation [17]. Mechanistically, dsRNA is a toll-like receptor 3 (TLR3) agonist, a strong inducer of humoral- and cell-mediated immunity that is mainly generated through inflammatory cytokines [19]. dsRNA leads to cellular release of TNF-α and, as reported recently, of TNF-α and IFN-γ, the main drivers of the cytokine storm and cell death occurring in severe forms of COVID-19 [20]. Moreover, a dsRNA-activated protein kinase (PKR) has been found to play a key role in inflammatory signaling [21] including TNF-α secretion. Of particular interest in the context of the COVID-19, a recent report describes that the interferon-induced, dsRNA-actived antiviral enzyme OAS1 significantly contributes to the antiviral response against SARS-CoV-2 [22]. OAS1 recognizes short structures of SARS-CoV-2 RNAs [22]. Importantly, a genetic polymorphisms in OAS1 introduces a variable sensing of dsRNA and influences COVID-19 severity [22].

A close look at the description of COVID-19 preparations by different mRNA vaccine developers [15,23] reveals the explicit report of a low-level dsRNA impurity for one [15] and no mention of residual dsRNA for another [23]. The presence of dsRNA, even at low levels, raises questions about its possible favorable and unfavorable consequences, bearing in mind that a new, improved technique to remove dsRNA has been reported recently [16]. This improved mRNA purification method, hardly transposable on an industrial scale, consists in the adsorption of dsRNA contaminants to cellulose and could work better than chromatographic methods of removing dsRNA from IVT mRNA samples.

Regarding the consequences of the presence of dsRNA (Figure 1), on the one hand, dsRNA could provide an advantage (intrinsic adjuvant) by inducing a high immunological response, which is obtained with COVID-19 mRNA vaccines. However, this remains an hypothesis and there are no dedicated studies evaluating a possible benefit of dsRNA in mRNA vaccines conferring an added protection against SARS-CoV-2. On the other hand, dsRNA could boost the induction of some uncontrolled and potentially detrimental immune-inflammatory reactions, such as myocarditis (Figure 1). This leads to the next unavoidable question: can the presence of dsRNA in mRNA vaccines, even at low concentration, explain some of the undesired effects? In the case of myocarditis, it should be noted that when packaged in lipid nanoparticles, dsRNA is preferentially transferred to phagocytic monocytic-derived cells, such as macrophages and dendritic cells, which are key actors in immunity [24]. Recent studies indicate that precursors of dendritic cells patrol the blood and communicate with immature dendritic cells residing in peripheral tissues such as the kidneys, skin and myocardium. Dendritic cells trigger immune responses in lymphoid tissues upon early sensing of infectious pathogens. Globally, dendritic cells form a sentinel network that modulates immune responses with the distinct ability to produce protective immunity or tolerance to self. Dendritic cells also play a major role in the pathophysiology of inflammatory diseases. As a result, the uptake of dsRNA by dendritic cells is suspected of triggering immune reactivity and inflammatory reactions.

Cardiovascular toxicity risks may be a concern with cancer therapies like radiotherapy [25] and chemotherapy by anthracyclines [26]. Fulminant cases have been reported with immunotherapy by checkpoint inhibitors [27]. The proinflammatory role of dsRNA, even at low concentration in mRNA COVID-19 vaccines, can constitute a true risk for myocarditis. This risk should thus be considered when prescribing vaccination in cancer patients who have been treated or are under an anticancer treatment that threatens the heart.

## Future perspective

Overall, COVID-19 vaccination has led to highly favorable results that exceeded what many had hoped for. Clinical trials are now underway to test second-generation vaccines in an effort to contain the spread of emerging SARS-CoV-2 variants [28]. Nevertheless, vaccine efficacy is only one aspect of the global control of the pandemic, other scientific, social and political questions need to be addressed concerning issues relating to doses, schedules and vaccine hesitancy. Consequently, even though vaccines clearly represent a way to end the COVID-19 pandemic, information regarding their risks must be communicated transparently to maintain trust [29]. This article considers and argues that the low levels of dsRNA present in COVID-19 mRNA vaccine preparations could be, alone or in addition to other predisposing factors, at the origin of the still unexplained cases of more or less severe myocarditis following vaccination. However, a relatively low level of clinical evidence is currently available in this context to be taken as hypothesis-generating. It is thus legitimate to expect in a near future more clinical knowledge and experimental investigations into the double-edged immunological and proinflammatory effects of dsRNA found in COVID-19 mRNA vaccines. Targeted investigations should be undertaken at the experimental level to determine the true impact of dsRNA vaccine contamination with appropriate dose-response analyses using pathologically relevant preclinical models. A comparison between dsRNA level in a given COVID-19 mRNA vaccine and the respective incidence of myocarditis would be welcome.

The quest for a better understanding of all aspects of COVID-19 vaccines should not be considered as mistrust, but as a legitimate claim for greater transparency and for optimizing basic expertise given the expanding range of available COVID-19 vaccines. Industrial partners currently need to increase COVID-19 vaccine production capacity. There is also the clear necessity to adapt future vaccine development to the emergence of SARS-CoV-2 variants [30,31]. Above all, the priority will be to maintain the intrinsic quality of vaccines at the highest level [31]. With the application of the third dose of COVID-19 vaccine, making the vaccinated population to become much larger than in the initial Phase III registration trials, unexpected security findings may still arise, as was the case with the thromboembolism of unclear origin suspected to be linked with the Oxford-AstraZeneca COVID-19 vaccine [32]. It is certain that confidence in COVID-19 vaccination must remain high, but a deeper knowledge of these vaccines and consideration of every detail, like this herein discussed presence of dsRNA impurity in mRNA vaccines, will help to elucidate the origin of unwanted effects and prevent their possible occurrence.

**Executive summary**

**Background**

- Candidate vaccines against severe acute respiratory syndrome coronavirus 2 have been developed at a particularly accelerated rate with remarkable efficacy.

**The occurrence of myocarditis under COVID-19 vaccination**

- The unanticipated occurrence of myocarditis under COVID-19 mRNA vaccination has been reported.

**Double-strand RNA as an impurity in mRNA vaccines**

- Double-strand RNA (dsRNA) appears to be a low-level impurity present in COVID-19 mRNA vaccines.

**dsRNA and immune-inflammatory reactions**

- dsRNA is a strong inducer of immune-inflammatory reactions.
- dsRNA could be hypothetically suspected to trigger the induction of myocarditis among other possible factors.
